# Editorial: New perspectives in the treatment of myasthenia gravis

**DOI:** 10.3389/fneur.2024.1418631

**Published:** 2024-06-11

**Authors:** Elena Cortés-Vicente, Bettina Schreiner, Francesco Saccà, Pushpa Narayanaswami

**Affiliations:** ^1^Unitat de Malalties Neuromusculars, Hospital Santa Creu i Sant Pau, Barcelona, Spain; ^2^Department of Neurology, University Hospital Zurich, Zürich, Switzerland; ^3^Department of Neurosciences, Reproductive and Odontostomatological Sciences, School of Medicine and Surgery, University of Naples Federico II, Naples, Campania, Italy; ^4^Beth Israel Deaconess Medical Center/Harvard Medical School, Boston, MA, United States

**Keywords:** myasthenia gravis, treatment strategy, new treatments, thymectomy, rituximab, complement inhibitors, neonatal Fc receptor antagonist

Myasthenia gravis (MG) is a chronic autoimmune disorder characterized by fatigable muscle weakness. The disease is very heterogeneous in terms of clinical manifestations depending on the muscle group involved, which includes the ocular, bulbar, limb and respiratory muscles. It is also complex from an immunological point of view. Anti-acetylcholine receptor antibodies are the most common, but anti-MuSK, anti-LRP4 or even seronegative patients can also be found. In approximately 5% of patients, no antibodies are detected (seronegative MG). Thymic abnormalities are frequent, mainly hyperplasia or thymoma, but there are patients with a normal thymus. Treatment includes acetylcholinesterase inhibitors, immunosuppressive and immunomodulatory drugs and thymectomy. Early diagnosis and appropriate management are essential to optimizing outcomes and quality of life for individuals with this condition.

In recent years our understanding of the physiopathology of the disease has led to new treatment strategies. The anti-CD20 monoclonal antibody rituximab was the first treatment used in the early 2000. In 2017, the first complement inhibitor, which prevents antibody-mediated complement activation and damage at the neuromuscular junction was approved for clinical use. Subsequently, these were followed by neonatal Fc receptor (FcRn) antagonists, which induce the catabolism of IgG (including pathogenic MG autoantibodies) by lysosomal degradation and reduce its extracellular concentration. This Research Topic aims to provide an overview of these emerging treatment strategies for MG. The authors discuss the underlying pathophysiological aspects of these agents. Clinical trials and observational studies are reviewed, describing the efficacy outcomes and safety profile of these drugs. These drugs are a new hope for MG patients, especially for drug-refractory patients. In parallel, a case report showing a refractory patient in myasthenic crisis and her response to efgartigimod is included. Myasthenic crisis patients are not usually included in clinical trials, and information about treatments that are useful to this subset of patients is important.

This Research Topic includes original research. Two articles studied the current treatment strategies to improve MG. The first described the therapeutic and prognostic features of MG patients followed in a tertiary neuromuscular disease center in Turkey. Interestingly, they found that the use of corticosteroids was more common in patients younger than 50 years, and the use of non-steroidal immunosuppressant drugs was more common over the age of 50. In keeping with this, the second study evaluates treatment modalities for early-onset (< 50 years of age) and late-onset MG (≥50 years of age). Although similar strategies and treatment-related adverse events were found, corticosteroid-related adverse events appeared to differ between groups, with hypertension, hypercholesterolemia, diabetes mellitus and malignancies being more common in late-onset MG patients. As MG can occur at any age, these findings may help in the selection of treatment options for our patients.

Thymectomy has been used to treat MG patients both with and without thymoma. However, new, limited surgical techniques have increased in popularity in recent years. A study showing the benefits of video-assisted thoracoscopic thymectomy (VATS) is included in this Research Topic. Patients undergoing VATS thymectomy had lower rates of intra-surgery and post-surgery complications, reduced morbidity, a shorter postoperative hospital stay, and a favorable impact on MG symptoms, both immediately post-surgery and in the long term, in addition to lower rates of local and distant thymoma recurrence when compared to patients undergoing sternotomy thymectomy. This work supports VATS thymectomy as the recommended surgical technique for thymic resection.

Another topic of great interest to neurologists treating MG patients is myasthenic crisis. This Research Topic includes a pilot study that used routine, cost-effective and widely available laboratory parameters related to inflammation and hemograms to identify potential risk factors for myasthenic crises. The results of this study provide proof of the concept that elevated basophil, neutrophil, leukocyte, and platelet counts may be associated with a higher risk of developing myasthenic crisis in MG patients. These potential biomarkers, together with clinical data, may help to individualize treatment strategies.

Finally, a bibliometric analysis of the characteristics of MG publications over the last 20 years was included by mapping the scholarly contributions of various countries or regions, institutions, journals, and authors in the field of MG. The close collaboration between countries and institutions was remarkable. This study also explored future trends and prospective directions, emphasizing individualized treatment based on subtypes, novel immunotherapeutic approaches, and thymectomy, all of which are featured topics in the current Research Topic.

Overall, this Research Topic aims to improve knowledge of current and emerging therapies for the treatment of MG, while stimulating research into unanswered questions. The new drugs are likely to change the current strategy for treating MG patients. Moreover, our increased knowledge of physiopathology will improve the selection of therapies for each subgroup of MG patients. When, how, and who to treat with each therapy is the burning question- we are closer to finding the answer.

## Author contributions

EC-V: Writing—original draft, Writing—review & editing. BS: Writing—review & editing. FS: Writing—review & editing. PN: Writing—review & editing.

